# Urban spatial structures from human flow by Hodge–Kodaira decomposition

**DOI:** 10.1038/s41598-022-15512-z

**Published:** 2022-07-04

**Authors:** Takaaki Aoki, Shota Fujishima, Naoya Fujiwara

**Affiliations:** 1 Faculty of Education, Kagawa University, Takamatsu, 760-8521 Japan; 2 Graduate School of Economics, Hitotsubashi University, Tokyo, 186-8601 Japan; 3 Graduate School of Information Sciences, Tohoku University, Sendai, 980-8579 Japan; 4 PRESTO, Japan Science and Technology Agency, Kawaguchi, 332-0012 Japan; 5 Center for Spatial Information Science, the University of Tokyo, Kashiwa, 277-8568 Japan; 6 Institute of Industrial Science, the University of Tokyo, Tokyo, 153-8505 Japan; 7 Tough Cyberphysical AI Research Center, Tohoku University, Sendai, 980-8579 Japan

**Keywords:** Applied mathematics, Complex networks, Civil engineering

## Abstract

Human flow in cities indicates social activity and can reveal urban spatial structures based on human behaviours for relevant applications. Scalar potential is a mathematical concept that, when properly applied, can provide an intuitive view of human flow. However, the definition of such a potential in terms of the origin-destination flow matrix and its feasibility remain unresolved. In this case, we use Hodge–Kodaira decomposition, which uniquely decomposes a matrix into a potential-driven (gradient) flow and a curl flow. We depict the potential landscapes in cities resulting from commuting flow and reveal how the landscapes have either changed or remained unchanged by years or methods of transportation. We then determine how well the commuting flow is described by the potential, by evaluating the percentage of the gradient component for metropolitan areas in the USA and show that the gradient component is almost 100% in several areas; in other areas, however, the curl component is dominant, indicating the importance of circular flow along with triangles of places. The potential landscape provides an easy-to-use visualisation tool for showing the attractive places of human flow and will help in a variety of applications such as commerce, urban design, and epidemic spreading.

## Introduction

Human mobility is a vital social activity in our society that is relevant to various applications in commerce, urban design, marketing, and economics while also being involved in the spreading of diseases such as COVID-19. Mobility data have long been collected through person-trip surveys, but currently, they are also collected through mobile phone tracking. The person-trip survey data are not real time (typically reported annually or decennially); however, they are well-organized into separated journeys based on the purpose of trips, transportation methods, and other valuable properties that are difficult to obtain explicitly by tracking mobile devices.

**Figure 1:**
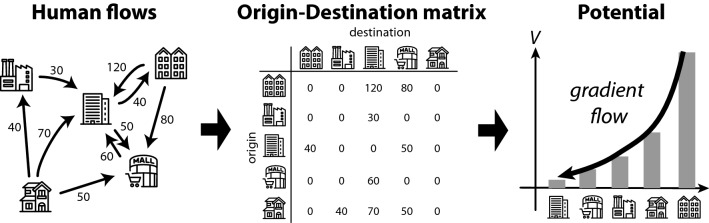
Origin-destination matrix and the concept of the potential of human flow.

These human mobility data are typically aggregated as an *origin-destination (OD) matrix* (Fig. 1), which describes how many people are moving from one location (origin) to another (destination). Thus, the mobility data characterises the relationships between places based on human behaviour and is expected to reveal the places that attract human flow and their basins. Such information tells us the centres and limits of cities and unfolds the actual shapes of cities, which dynamically change according to years, transportation methods, and movement restrictions. They, in turn, aid location decision-making for commercial or public buildings, the optimisation of transportation systems, urban planning by policymakers, and measures for movement restrictions to reduce the spread of COVID-19.

We consider the *scalar potential* of human flow to reveal the spatial structure of cities. Potential is a popular mathematical concept used in various scientific fields, ranging from physics to economics. In the context of our study, it is defined as a function of location, and its gradient yields the net movement of people between locations. Such a potential landscape provides an intuitive perspective of human flow by analogously representing water flowing from a higher place to a lower place. Furthermore, it reduces the relational flow data to location-level statistics that are ready to be shown on a map. The map allows us to easily identify the sinks and sources of human flow, as illustrated in Fig. 1. A sink of human flow indicates attractive places. Potential landscape can visualise the urban structure behind massive data of human mobility and utilise it for relevant applications, if successfully introduced.

However, it is not obvious how to introduce the potential to human flow. Unlike an electromagnetic field, human flow is not described by a two-dimensional vector field, but as an OD matrix. Furthermore, it is an open question whether human flow can be effectively described by a potential in the first place, according to Helmholtz’s theorem. In literature, the OD matrix is converted into a 2D vector field by averaging all trips from each location^1^, focusing solely on the motion of the centre of mass rather than the motions of individuals. The resultant vector field was found to be almost irrational, and a scalar potential was introduced. However, this aggregation discards the place-to-place information of the original data. We demonstrate through benchmark tests using synthetic data that using the previous method it is difficult to identify the number of centres and their areas expressed in the given data.

Another approach is to define a potential^2^ or attractiveness^3^ using the gravity model^4–6^, which is a well-known model for human flow. Several residential and economic datasets have been used to evaluate these measures^7,8^. These measures, however, are specific to the assumed model and are not calculated from the OD matrix data.

Here, we provide a straightforward introduction of a potential to the OD matrix by applying the Hodge–Kodaira decomposition of graph flow^9–13^. As described in the "Methods" section, human flow is uniquely decomposed into two distinct flows: a potential-driven (gradient) flow and a circular flow. The potential at each place is directly and easily calculated from a given OD matrix without any model assumptions and calibration parameters. The potential is interpretable: it refers to the difference between incoming and outgoing flux of people. Furthermore, the decomposition allows us to determine how well the potential describes human flow by evaluating the percentage of the gradient component. We observe that the circular component in human flow is not always negligible. This is in contrast to the previous study that treated the circular flow as noise^1^.

Following an overview of the decomposition method, we validate potential extraction methods using benchmark tests for conceptual situations. Then, we depict the potential of the commuting flow in London for several different transport methods and show the evolution of the potential landscape over 30 years in Tokyo. We then study the percentage of the gradient component in metropolitan areas in the USA. Finally, we discuss the practical implications of the potential and limitations of the proposed method.

## Results

### Overview of Hodge–Kodaira decomposition to an OD matrix

In this section, we review Hodge–Kodaira decomposition as it applies to an OD matrix. We assume that people can travel between any pair of locations. Technically, this assumption corresponds to the case of complete graphs in the method’s general description (see Methods for the details).

First, we consider the net flow of movement from a given OD matrix *M* as when 150 persons move from location *i* to another location *j* and 50 people move in the opposite direction, we consider the net movements of 100 persons from *i* to *j*. The net flow is given by

1 $$\begin{aligned} A = M - M^{\intercal }, \end{aligned}$$

where $M^{\intercal }$ denotes the transpose of *M*. The matrix *A* is skew-symmetric, that is, $A_{ij} = - A_{ji}$, and is possibly described by combinatorial gradient of a potential *s*, given by

2 $$\begin{aligned} (\text {grad}\, s)(i, j) = s_j-s_i. \end{aligned}$$

Then, we define the optimisation problem for potential *s*:

3 $$\begin{aligned} \min _s \Vert \text {grad}\ s - A \Vert _2 = \min _s \left[ \sum _{i,j} \left[ (s_j - s_i) - A_{ij} \right] ^2 \right] . \end{aligned}$$

According to the combinatorial Hodge theory^11^, the space of net flow ${\mathcal {A}}$ is orthogonally decomposed into two subspaces:

4 $$\begin{aligned} {\mathcal {A}} = \text {im}(\text {grad}) \oplus \text {im}(\text {curl}^*), \end{aligned}$$

where $\text {curl}$ is the combinatorial curl operator and $\text {curl}^*$ is its adjoint operator. Thus, the optimisation problem is equivalent to an $l_2$-projection of *A* onto im(grad), and the minimal norm solution is simply given by

5 $$\begin{aligned} s_i = -\frac{1}{N} \text {div} A = - \frac{1}{N} \sum _{j=1}^N A_{ij}, \end{aligned}$$

where $s_i$ is the potential at the *i*th location and *N* is the number of locations. Using equation (1), the potential is rewritten as

6 $$\begin{aligned} s_i = \frac{1}{N} \left( \sum _{j \ne i}^N M_{ji} - \sum _{j \ne i}^N M_{ij} \right) . \end{aligned}$$

Note that $s_i$ is negative potential ($s_i=-V_i$). This means that we see more trips from a location with low potential to another with high potential.

The matrix *A* is orthogonally decomposed into gradient and curl components. To determine how well the potential describes human flow, we define the percentage of gradient component as:

7 $$\begin{aligned} R^2 =\frac{\sum _{i,j} \left[ (\text {grad}\, s)(i, j) \right] ^2}{\sum _{i,j} A_{ij}^2} = 1 - \frac{ \sum _{i} \sum _{j \ne i} \left( A_{ij} - (s_j - s_i) \right) ^2 }{\sum _{i} \sum _{j \ne i} \left( A_{ij} \right) ^2 }. \end{aligned}$$

This quantity is known as the ‘coefficient of determination’ in statistics. It is a reasonable choice for assessing the explanatory power of the potential, which is determined using orthogonal projection and is similar to ordinary least squares. In the following, we will show the values of $R^2$ as percentages by multiplying 100.

### Benchmark test using synthetic OD matrix

Before investigating the potential of human flow in real cities, we validate potential extraction methods by benchmark tests for which the OD matrix was synthetically derived from a given potential ${\bar{V}}$:

8 $$\begin{aligned} {\bar{M}}_{ij} = [ - ({\bar{V}}_j - {\bar{V}}_i) ]_{+}, \end{aligned}$$

where $[x]_+ = \max (0,x)$ is a rectifier to ensure positive trips. For the synthetic OD matrix, we treat ${\bar{V}}$ as the “ground truth” of the potential. We validated the extracted potential ${\hat{V}}$ from the synthetic OD matrix ${\bar{M}}$ by comparing with this true potential and calculated the mean squared error (MSE):

9 $$\begin{aligned} \text {mean squared error} = \frac{1}{N} \sum _{i = 1}^N ({\hat{V}}_i - {\bar{V}}_i)^2. \end{aligned}$$

In the comparison, the potential is standardised such that its maximum value matches with the reference value ($V=0$).

**Figure 2:**
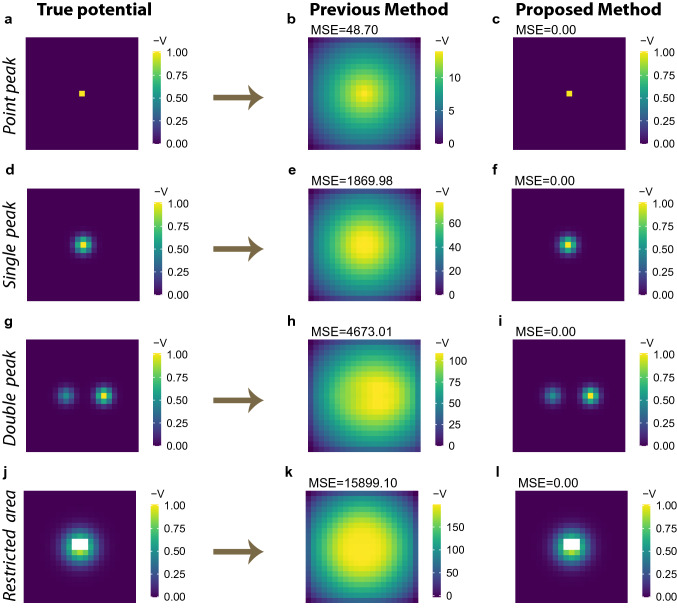
Potential identification for conceptual situations. (**a–c**) *point peak* situation represents an artificial monocentric city. There is an attractive location at the centre, to which people move from all other locations (**a**). Given an OD matrix generated by this situation, the potential estimated by the previous method^1^ is shown in panel (**b**) and that by the proposed method is shown in panel (**c**). (**d-f**) *single peak* situation is similar to the *point peak* situation, but the central place has some spatial extents (**d**). The potential is estimated by the previous method (**e**) and the proposed method (**f**), respectively. (**g-i**) *double peak* situation represents a polycentric city, in which there are two attractive places (**g**). The potential is estimated by the previous method (**h**) and the proposed method (**i**), respectively. (**j-l**) In the *restricted area* situation (**j**), the potentials at some locations (white cell) are not available (*NA*), because they are, for example, a restricted area or a lake. These *NA* locations cannot be the origin or destination of flow. The potential is estimated by the previous method (**k**) and the proposed method (**l**), respectively.

Figure 2 shows the benchmark results; the left panels show typical urban structures represented by the potential ${\bar{V}}$ and the middle and right panels show those obtained from the previous method in^1^ and the proposed method, respectively. This indicates visually whether each of the two methods recovers the “true” structures correctly from the synthetic OD matrix.

The first *point peak* situation represents an ideal monocentric city (Fig. 2a). There is an attractive location at the centre, and the potential of the other locations is equal to the reference value (${\bar{V}}$ = 0). In this situation, people gather at the single point at the centre, and the flow is seen as a star network. Under this condition, the potential ${\hat{V}}$ extracted by the previous method in^1^ is peaked at the same centre but has a broader distribution (Fig. 2b). This suggests that the area near the centre is differentiated from more peripheral areas by the potential ${\hat{V}}$. This is inconsistent with the ground truth of the city structure, in which all the locations are identical except for the central point.

Next, in the *single peak* situation (Fig. 2d), the attractive place has some spatial extents. In this condition, the potential ${\hat{V}}$ extracted by the method in^1^ still has a wider distribution (Fig. 2e); therefore, the central area by ${\hat{V}}$ appears larger than its actual size.

The *double peak* situation represents a polycentric city (Fig. 2g). There are two attractive places that draw people from the other locations. The place on the right-hand side is more attractive than the one on the left-hand side, as shown by their potential values; thus, the right-hand side is the main centre, and the left-hand side is a sub-centre. The extracted potential ${\hat{V}}$ in^1^ has only a single-peak broad distribution (Fig. 2h); thus, it is difficult to observe the clear polycentric structure. This misidentification is caused by the conversion process from the place-to-place flow to a 2D vector field as described in equation (21) in the "Methods" section. In the Tokyo metropolitan area, for example, *Kawasaki* city is known as a sub-centre to which people commute^14^. At the same time, many residents in the area commute to the largest central area around *Chiyoda* city. This is a common situation in a metropolitan area, which is often defined as an urban centre and its commuter hinterland, such as core-based statistical areas in the US or travel-to-work areas in the UK. In this case, the averaged vector at *Kawasaki* city will be directed toward the largest centre, and the sub-centre is hidden. The potential shown in^1^ for the Tokyo metropolitan area has no peak at *Kawasaki* city and other known sub-centres; therefore, the previous method would be unsuitable for discussing polycentric structures within metropolitan areas.

The *restricted area* situation in Fig. 2j is similar to the *single peak* situation, with some locations near the centre labelled as *NA*. Here, *NA* indicates that the potential is undefined because the location is a non-land cell, such as a river, lake, or sea, or a restricted area by law. Some historical cities, for example, have developed around palaces or castles, which frequently had restricted areas. The *NA* locations cannot be the origin or destination of a flow. The potential obtained by the previous method spreads over the *NA* locations (Fig. 2k), which has also been observed in real cities^1^. Furthermore, it identifies those locations as a part of a central area. The potential at a non-land cell or restricted area would be difficult to interpret.

These observed deviations from the true potential ${\bar{V}}$ are quantitatively measured in terms of mean squared error. Although the large errors obtained via the previous method are partly caused by inconsistencies in the generation process of the OD matrix by equation (8), the concepts of the investigated situations are generic and independent of the specific equation. The conversion process from the place-to-place flow to the 2D vector field, which is intrinsic to the method, discards the essential information of the urban structures represented in the given flow.

In contrast to the previous method, the proposed decomposition method perfectly recovers the true potentials without any error, in every situation (Fig. 2c,f,i,l). It does not assign any potentials to non-land cells or restricted areas, that is those labelled as *NA*. Therefore, in this benchmark test, we can identify urban structures, such as the location of a city centre, its area, or the number of centres, as represented by the potential. It should be noted that the synthetic flow only contains the gradient component, which is generated by a given potential, and the potential can be perfectly identified by the method. However, actual human flow could have another component (curl flow) as described in equation (4), which is not explained by the potential.

Furthermore, it should be noted that these benchmark examples are not unduly detrimental to the previous method. It is actually advantageous: the method requires flows to be provided at grid points, as shown in this benchmark. Human flow datasets, on the other hand, are typically aggregated by administrative units in survey-based collections or Voronoi polygons of cell towers in call detail records (CDRs) of mobile phones, necessitating some resampling treatments to grid points. By contrast, the proposed method is applicable to the OD matrix aggregated by any shape of the geographical zones.

### Potential landscapes in cities

As a first demonstration, we show the potential landscape in Greater London in 2011, using a person-trip dataset from home to workplace. The OD matrix shows the number of commuters aggregated by the middle layer super output area (MSOA) in the 2011 census (see "Methods" for details). The trips were categorised based on the method of travel used for the longest part by distance. We first show the potential by all the methods and then by specific transport methods. This allows us to investigate the urban structures from different viewpoints through transport methods.

**Figure 3:**
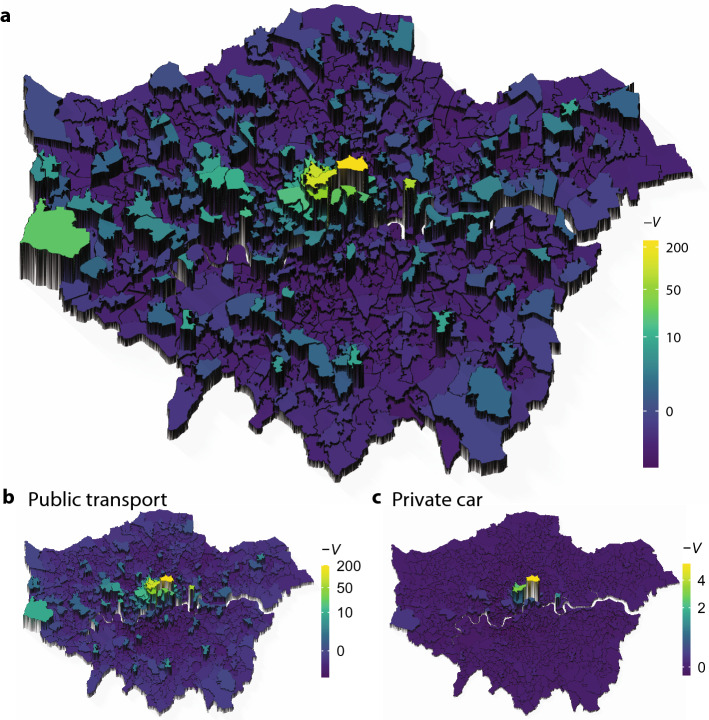
Negative potential $-V$ of the home-work trips in London. (**a**) The potential at a place is indicated by its colour and its height. (**b,c**) The potentials are depicted for the selected trips by specific transport methods.

Figure 3 depicts the negative potential $-V_i (=s_i)$ of the decomposed gradient flow. The potential has the largest peak at “City of London 001”, literally the centre of London. Its neighbouring areas, such as “Westminister 018” and “Westminister 013”, also have large potentials. Another peak, that is, a local maximum in the potential landscape, is seen at “Tower Hamlets 033”, and there are small peaks outside the central area of London. Most other areas are characterised by a relatively lower potential by $-V$, serving as the sources of commuters to the centres.

The flows selected by specific transport methods provide another picture of potential landscapes in London. The potential for public transportation (Fig. 3b) is similar to that of all methods. By contrast, the potential for private cars (Fig. 3c) becomes a *single-centre* city than *multi-centre*: a few locations still have higher potential, and the other locations have very low potentials without small peaks. In addition, the potential amplitude is smaller than that for other cases, reflecting the volume of commuters (public transport = 1.6 million trips, private car = 45.2 thousand trips).

Next, we demonstrate how the urban structures in Tokyo have either changed or remained unchanged over 30 years in terms of the time evolution of the potential landscape. We used the commuter datasets of successive person-trip surveys from 1988 to 2018 in the Tokyo metropolitan area (see "Methods" for details).

**Figure 4:**
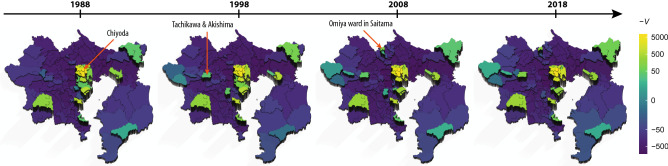
Time-evolution of the potential landscape in Tokyo metropolitan area. The potentials are obtained from the home-work trips of successive surveys over 30 years in the Tokyo metropolis and surrounding provinces. Each zone is basically equal to a municipal district.

Figure 4 shows that, over 30 years, *Chiyoda* city—the Imperial Palace and its surrounding areas—has been at the top of the potential. The city is known as the economic and political centre of Japan: it houses the headquarters of major enterprises, government institutions, and the Tokyo Central Railway Station. Its neighbouring cities, such as *Minato*, *Chuo*, *Shinjuku*, and *Shibuya*, have occupied the top five ranks, by potential, over the years (Supplementary Table S1), and formed the largest stable peak in the Tokyo metropolitan area. Several small, steady peaks were observed outside the central area (e.g. *Yokohama*, *Chiba*, *Kawasaki*, and *Atsugi* cities). In contrast to these steady peaks, new peaks appeared in *Tachikawa* and *Akishima* cities after 1998 and at the *Omiya* ward in *Saitama* city after 2008. These small peaks correspond to the business cores envisioned by the fourth National Capital Regional Development Plan in 1986^15^, which aimed at multi-nucleated urban structures to avoid over-concentration in the Tokyo central area.

### How much of the percentages of human flows are represented by potential?

To answer this question, we evaluated the percentage of the gradient component $R^2$ defined in equation (7) for many cities and examined its distribution. We used the person-trip dataset for the metropolitan areas in the USA in 2018 (see “Methods” for details). In this dataset, the metropolitan area is given by core-based statistical area (CBSA), a standard definition of the geographical area of cities. The dataset covers almost all CBSAs in the USA and compares the percentage $R^2$ across many metropolitan areas.

**Figure 5:**
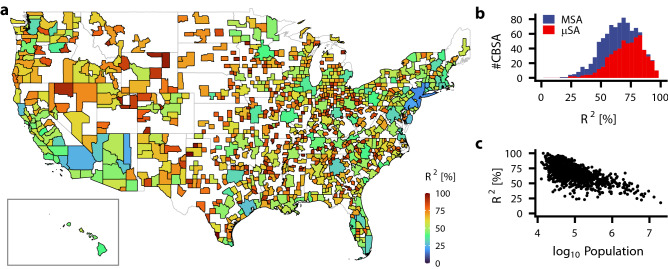
Percentage of gradient component $R^2$ in human flow. (**a**) Percentage $R^2$ in home-work trips for each Core-based statistical area (CBSA) in 2018. The inset shows the CBSAs in Hawaii. (**b**) Histogram of percentage $R^2$. CBSAs are classified into metropolitan statistical areas (MSAs) and micropolitan statistical areas ($\mu$SAs). (**c**) Percentage $R^2$ is plotted against the population of the CBSAs.

Figure 5a shows the percentage $R^2$ for each metropolitan area in the USA. The percentage $R^2$ varies widely among the areas: the minimal percentage $R^2$ = 17.72% was in *New York-Newark-Jersey City, NY-NJ-PA*, while the maximum was 99.98% in *Zapata, TX*. The distribution has a mean of $\mu$ = 66.2% and standard deviation $\sigma$ = 15.3% (Fig. 5b). In CBSAs, metropolitan statistical areas (MSAs) tend to have a lower percentage than micropolitan statistical areas ($\mu$SAs). Thus, the percentage $R^2$ is plotted against the population (Fig. 5c), showing that the percentage $R^2$ tends to decline for larger populations. In addition, the percentage $R^2$ was changed by the transport methods in the London case (Supplementary Table S2) and by years in the Tokyo case (Supplementary Table S3).

## Discussion

In this study, we introduced a potential for the OD matrix by Hodge–Kodaira decomposition and depicted the potential landscape in cities. In London, the largest peak of the potential landscape, that is, the most attractive centre of the flow, is located at “City of London 001”. The landscape could give a different view of urban structures by the transportation method. In the Tokyo metropolitan area, the time evolution of the potential over 30 years revealed how Tokyo had either changed or remained unchanged from the viewpoint of human flow. We observed that the largest peak was stably located in *Chiyoda* city, which is the central area of Tokyo. Other peaks were observed in suburban business cores, confirming the development of the multi-nucleated urban structures envisioned in the 1986 national development plan. These business cores are also known as “edge cities” of Tokyo, which are dynamically organised^14,16,17^. In fact, it is clearly shown that some cores have emerged over the years as new peaks in the potential landscape.

We first discuss the practical meaning of the potential we introduced. According to equation (6), the potential is clearly interpreted as the difference between incoming and outgoing flux of people. In other words, a location with a greater incoming flow from other locations and a smaller outgoing flow to other locations becomes a location with a higher potential. The total balance of incoming and outgoing flux determines the attractiveness of a location in terms of potential *s*.

The potential of human flow also incorporates the importance of circular flow in cities. We evaluated how well the potential describes human flow in metropolitan areas in the US by using the percentage of the gradient component. We found that the percentage is not always 100% and not a universal value but highly variable among the areas. For several areas, the gradient component is more dominant, with a high percentage $R^2$, while in other areas, the other component (curl component) is dominant. This variation reflects the differences in human flow across the areas and raises new questions of when and why human flow is well described by the potential. Furthermore, the curl component tends to be dominant for large cities, indicating the importance of circular flow in net movements of people. The curl is defined for triplets of locations as described in the "Methods" section. By contrast, human flow has been discussed in terms of paired locations: origin and destination. The circulation along triangle places addresses a new aspect of human flow with another question: What drives the circular flows in populated areas? The decomposition method opens up new research avenues in human mobility and urban structures.

The limitation of the proposed method should be noted. The potential is based on the rigorous mathematical definition of the OD matrix and does not require any model assumptions and any additional datasets. Conversely, the analysis in this study does not consider several factors assumed in spatial interaction models, such as the gravity model^4–6^ or radiation model^18^. In particular, the distance deterrence on human mobility is not considered. This could impose limitations on a native application to a dataset at the country level, where distance critically matters. Thus, it is appropriate to apply decomposition to the human flow dataset within cities or narrow regions. Otherwise, a distance-weighted function can be integrated into the decomposition, as described in the “Distance deterrence effect” section in Supplementary Information.

In summary, the potential landscape by Hodge–Kodaira decomposition provides an intuitive perspective of human flow by its gradient flow from a higher place to a lower place. The landscape allows us to understand the spatial structure of cities based on human movements rather than administrative circumstances and to study the dynamic changes in the spatial structure under different conditions. For example, we can study whether the global increase in remote workers due to the COVID-19 pandemic is alleviating over-concentration of population in city centres by checking the emergence of new potential peaks in suburbs or the decline of pre-existing ones. The method provides an easy-to-use visualisation tool to show the places attracting human flow and will aid relevant applications in commerce, urban design, and epidemic spreading.

## Methods

### Hodge–Kodaira decomposition to OD matrix

The origin-destination (OD) matrix *M* is a square matrix that represents the number of trips from origin *i* to destination *j* by its elements $M_{ij}$. Any square matrix *M* is uniquely decomposed into a symmetric and a skew-symmetric matrix,

10 $$\begin{aligned} M = \underbrace{\frac{1}{2} \left( M + M^{\intercal } \right) }_{\text {symmetric }} + \underbrace{\frac{1}{2} \left( M - M^{\intercal } \right) }_{\text {skew-symmetric }}, \end{aligned}$$

where $M^{\intercal }$ is the transpose of *M*. The symmetric part can be further decomposed into diagonal and off-diagonal elements. The former represents a self-loop flow at each location. The latter part is the bidirectional circulation of people between two locations. Although investigating these symmetric elements would be interesting, we will concentrate on the skew-symmetric part because it may be described by the gradient flow. In this study, we analyse *A* ($= M - M^{\intercal }$) by multiplying the skew-symmetric part by 2. The matrix *A* represents the net movement of human flow, by removing the self-loop and bidirectional circulations.

We decompose the net flow *A* by the Hodge–Kodaira decomposition. The decomposition is, in general, defined for an undirected graph $G({\mathcal {V}},E)$, where the vertex set is ${\mathcal {V}}$ and the edge set is *E*. The element $A_{ij}$ represents the flow at the edge^11^. According to^11^, the combinatorial gradient operator and combinatorial curl operator are defined as follows:

11 $$\begin{aligned} (\text {grad}\, s)(i, j)&= s_j-s_i \quad \text {for} \{i,j\} \in E , \end{aligned}$$

12 $$\begin{aligned} (\text {curl}\, A)(i, j, k)&= A_{ij} + A_{jk}+A_{ki}\quad \text {for} \{i,j,k\} \in T(E) , \end{aligned}$$

where *s* is a potential function, *T*(*E*) is the set of triangles in the graph, and $\{\{i, j, k\}: \{i, j\}, \{j, k\}, \{k, i\} \in E\}$. Using these operators, the space of edge flow ${\mathcal {A}}$ is orthogonally decomposed into three subspaces,

13 $$\begin{aligned} {\mathcal {A}}&= \underbrace{\text {im}(\text {grad})}_{\text {gradient flow}} \oplus \underbrace{\text {ker}(\Delta _1)}_{\text {harmonic flow}} \oplus \underbrace{\text {im}(\text {curl}^*)}_{\text {curl flow}}, \end{aligned}$$

where ker($\Delta _1$) = ker(curl) $\cap$ ker(div), and $\text {curl}^*$ is the adjoint operator of the curl. With a Euclidean inner product in the space ${\mathcal {A}}$, $\langle X,Y\rangle = \sum _{ \{i,j\} \in E} X_{ij}Y_{ij}$, we define an optimisation problem:

14 $$\begin{aligned} \min _s \Vert \text {grad}\ s - A \Vert _2 = \min _s \left[ \sum _{\{i,j\} \in E} \left( (s_j - s_i) - A_{ij} \right) ^ 2 \right] . \end{aligned}$$

This is equivalent to an $l_2$-projection of *A* onto im(grad). Then, the solution of the optimisation problem satisfies the following normal equation^11^:

15 $$\begin{aligned} \Delta _0 s = - \text {div} A, \end{aligned}$$

where $\Delta _0$ is the graph Laplacian of graph *G*, and the divergence is (div *A*)(*i*) = $\sum _{j \text { s.t. } \{i,j\} \in E} A_{ij}$. Potential *s* with the minimal norm is given by,

16 $$\begin{aligned} s = - \Delta _0^{\dagger } \text {div} A, \end{aligned}$$

where $\dagger$ denotes the Moore-Penrose inverse. Similarly, the vector potential $\Phi$ of curl flow is derived as

17 $$\begin{aligned} \Phi =(\mathrm {curl} \circ \mathrm {curl}^{*} )^{\dagger } \mathrm {curl} A. \end{aligned}$$

The OD matrix determines the edge flow for every pair of nodes, corresponding to a complete graph. It should be noted that no edge between *i*, *j* in graph *G* means that the flow $A_{ij}$ between them is undefined or unavailable, and does not mean zero movement, $A_{ij} = 0$. In this case of a complete graph, $\text {dim}(\text {ker} (\Delta _1) )$ = 0 holds, and the matrix *A* is decomposed into only two parts: gradient and curl flows. Furthermore, the scalar potential in (16) and the vector potential in (17) are simplified as follows:

18 $$\begin{aligned} s_i&= -\frac{1}{N}\sum _{j=1}^N A_{ij}, \end{aligned}$$

19 $$\begin{aligned} \Phi _{ijk}&= \frac{1}{N}(A_{ij} + A_{jk} + A_{ki}), \end{aligned}$$

where *N* is the number of nodes. Using these potentials, the net flow *A* is uniquely decomposed as

20 $$\begin{aligned} A_{ij}&= (\text {grad}\, s)(i, j) + (\text {curl}^*\, \Phi )(i, j) \nonumber \\&= (s_j - s_i) + \sum _k \Phi _{ijk}. \end{aligned}$$

### The method used in the benchmark test

We briefly describe the method proposed in a related work^1^, which we used in the benchmark test in Fig. 2. First, the OD matrix $M_{ij}$ is converted into a 2D vector field $\mathbf {W}_i$ by averaging all trips from each location *i*:

21 $$\begin{aligned} \mathbf {W}_i = \sum _{j \ne i} \frac{M_{ij}}{ \sum _j M_{ij}} \mathbf {u}_{ij}, \end{aligned}$$

where $\mathbf {u}_{ij}$ is the unit vector from location *i* to location *j*.

Next, the empirical potential *V* is numerically computed on a square grid. For a cell *i* with indices ($\alpha ,\beta$) on the grid, the equation $- \nabla V_i = \mathbf {W}_i$ is discretised by,

$$\begin{aligned} \frac{d V_i}{dx}&= \frac{V_{\alpha +1,\beta } - V_{\alpha ,\beta } }{\Delta x} = W^x_{\alpha ,\beta }, \\ \frac{d V_i}{dy}&= \frac{V_{\alpha ,\beta +1} - V_{\alpha ,\beta } }{\Delta y} = W^y_{\alpha ,\beta }, \end{aligned}$$

where $W^x$ and $W^y$ are *x* and *y* components of $\mathbf {W}$, respectively. Starting from one of the city bounding box corners with the boundary condition *V* = 0, the potential *V* at all the other cells are calculated by this discretisation formula. Different resultant potentials *V* can be obtained for each starting point (bounding box corner). We average them to calculate the outcome of the empirical potential *V* as proposed in the paper^1^.

### Datasets

#### London

We used a 2011 person-trip dataset, obtained from the UK Data Service^19^. This included typical one-way trips from home to work with no return trips. The OD matrix denotes the number of commuters aggregated by middle layer super output area (MSOA) in the 2011 census. The shapefile of the MSOAs is obtained from Office for National Statistics^20^. The dataset covers the MSOAs in England and Wales. In this paper, we selected only the trips among the MSOAs in Greater London. The resultant matrix contains 2.9 million trips between 983 MSOAs.

The trips in the dataset are categorised by main transport methods used for the longest part, by distance, and we selected the following two types of transport methods: “Public transport” includes the trips by underground, metro, light rail, tram, train, Bus, minibus, and coach. “Private car” includes the trips by driving a car, taxi, motorcycle, scooter, or moped, including their passengers.

#### Tokyo

We used datasets from successive person-trip surveys from 1988 to 2018 in the Tokyo metropolitan area^21^. The datasets were categorised according to the purpose of trips, and one-directional trips from home to the workplace were selected. The OD matrix denotes the number of commuters aggregated by middle-sized geographical zones. A middle-sized zone is essentially equivalent to a municipal district, with the exception that some zones in rural areas contain several districts. The zones have been altered by municipal mergers and dissolutions between 1988 and 2018, and the target regions of the surveys have been extended. We selected the areas covered by all surveys from 1988 to 2018 and have a surjective mapping from the zones in 2018, to make shapefiles before 2018 (The shapefile was available only for the last survey in 2018). Several peripheral areas in the Ibaragi, Chiba, Kanagawa, and Saitama provinces were excluded.

The resultant matrix contained 11.77 million trips among 121 zones in 2018, 11.74 million trips among 120 zones in 2008, 10.97 million trips among 114 zones in 1998, and 9.97 million trips among 106 zones in 1988.

#### Core-based statistical area (CBSA) in the United States

We used LEHD Origin-Destination Employment Statistics (LODES) datasets for 2018^22^. The datasets contain the number of jobs for each pair of residential places and workplaces at the census block level. We aggregated the data into the census tract level and analysed the OD matrix of commute trips for each core-based statistical area (CBSA) defined by the U.S. Office of Management and Budget^23^. The shapefiles of the census tracts were obtained from 2019 TIGER/Line shapefiles^24^.

There were 930 CBSAs in 2018, excluding those in Alaska and Puerto Reco^25^. CBSAs are classified into metropolitan statistical areas (MSAs) and micropolitan statistical areas ($\mu$SAs), depending on whether the population is larger than 50,000.

The population of a CBSA was computed by adding those of the counties that belong to the CBSA. County populations were taken from^26^.

## Supplementary Information

Supplementary Information.

## Data Availability

Person-trip datasets and the census data that support the findings of this study are publicly available, as noted in the "Methods" section.

## Code availability

The code is available in the GitHub repository at https://github.com/TakaakiAokiWork/HodgePotentialHumanFlow/.
