# Supplementary material for: Urban spatial structures from human flow by Hodge–Kodaira decomposition
Source: Sci Rep. 2022 Jul 4;12:11258. doi: 10.1038/s41598-022-15512-z (PMC9252991; doi:10.1038/s41598-022-15512-z)
Supplement: Supplementary file 1 — Supplementary Information. [file 41598_2022_15512_MOESM1_ESM.pdf]

Supplementary Information for
Urban spatial structures from human flow by Hodge-Kodaira decomposition

Takaaki Aoki,* **Shota Fujishima, Naoya Fujiwara**

*E-mail: takaaki.aoki.work@gmail.com.

Contents

1 Ranking of potential in the Tokyo metropolitan area from 1988 to 2018 1

2 Percentage of gradient components in London and Tokyo. 3

3 Distance deterrence effect. 3

1 Ranking of potential in the Tokyo metropolitan area from 1988 to 2018

Table S1 shows the top 20 zones by negative potential $-V$, using the commuter datasets of successive person-trip surveys from 1988 to 2018 in the Tokyo metropolitan area.

Over 30 years, *Chiyoda* city — the Imperial Palace and its surrounding areas — has been at the top of the potential. Its neighbouring cities, such as *Minato*, *Chuo*, *Shinjuku*, and *Shibuya*, had occupied the top five ranks by the potential over the years.

Some zones, such as *Naka* ward in *Yokohama* city, *Kawasaki* ward in *Kawasaki* city, and *Sumida* city, have declined in the rankings from 1988 to 2018. By contrast, some zones, such as *Tachikawa* and *Akishima* cities and *Omiya* ward in *Saitama* city, have risen in the rankings.

1988 Zone	$-V$	1998 Zone	$-V$	2008 Zone	$-V$	2018 Zone	$-V$
Chioda city	6808	Chioda city	5916	Chioda city	7170	Chioda city	6554
Chuo city	5251	Minato city	4952	Minato city	6580	Minato city	5367
Minato city	5164	Chuo city	4412	Chuo city	4454	Chuo city	3337
Shinjuku city	2854	Shinjuku city	2999	Shinjuku city	3088	Shinjuku city	2927
Shibuya city	1696	Shibuya city	2101	Shibuya city	2280	Shibuya city	2237
Taito city	1236	Taito city	1142	Shinagawa city	1317	Shinagawa city	1358
Naka ward, Yokohama city	849	Shinagawa city	1054	Koto city	992	Koto city	1221
Shinagawa city	665	Toshima city	875	Taito city	878	Bunkyo city	695
Toshima city	647	Naka ward, Yokohama city	793	Bunkyo city	824	Taito city	616
Kawasaki ward, Kawasaki city	633	Bunkyo city	783	Naka ward, Yokohama city	704	Nishi ward, Yokohama city	578
Bunkyo city	625	Koto city	658	Toshima city	682	Naka ward, Yokohama city	513
Nishi ward, Yokohama city	402	Nishi ward, Yokohama city	610	Nishi ward, Yokohama city	561	Atsugi city with Aikawa and Kiyokawa areas	327
Sumida city	322	Kawasaki ward, Kawasaki city	605	Chuo ward, Chiba city	372	Chuo ward, Chiba city	286
Atsugi city with Aikawa and Kiyokawa areas	191	Chuo ward, Chiba city	585	Kawasaki ward, Kawasaki city	330	Toshima city	272
Chiba city	183	Sumida city	343	Atsugi city with Aikawa and Kiyokawa areas	283	Mihama ward, Chiba city	217
Koto city	129	Atsugi city with Aikawa and Kiyokawa areas	207	Sumida city	233	Kawasaki ward, Kawasaki city	214
Meguro city	58	Yokosuka city	122	Mihama ward, Chiba city	137	Omiya ward, Saitama city	180
Ota city	47	Ota city	106	Omiya ward, Saitama city	116	Tachikawa and Akishima cities	117
Narita city with Tomisato and Sakae towns	37	Meguro city	89	Tachikawa and Akishima cities	42	Sumida city	85
Yokosuka city	35	Narita city with Tomisato and Sakae towns	85	Yokosuka city	39	Saiwai ward, Kawasaki city	80

Table S1. Top 20 zones of potentials in Tokyo metropolitan area from 1998 to 2018.

2 Percentage of gradient components in London and Tokyo.

The percentage R^2 was evaluated by the transport methods in the London case (Table S2) and by year in the Tokyo case (Table S3).

Transport Method	Percentage
All	52.7
Public transport	63.7
Private car	21.5

Table S2. Percentage R^2 of gradient component of home-work trips in London.

Year	Percentage
1988	42.4
1998	41.3
2008	40.8
2018	37.4

Table S3. Percentage R^2 of gradient component of home-work trips in Tokyo metropolitan area.

3 Distance deterrence effect.

Distance is obviously an important factor of human mobility. This factor has been considered as a *distance-deterrence function* $f(d)$ in spatial interaction models, such as the gravity model. We here integrate the function into the Hodge-Kodaira decomposition.

We rewrite the optimization problem in equation (3) as

$$\min_s \left[\sum_{\{i,j\}} \left[f(d_{ij})(s_j - s_i) - A_{ij} \right]^2 \right] = \min_s \left[\sum_{\{i,j\}} |f(d_{ij})|^2 \left[(s_j - s_i) - \frac{A_{ij}}{f(d_{ij})} \right]^2 \right] = \min_s \left[\sum_{\{i,j\}} w_{ij} \left[(s_j - s_i) - A^w_{ij} \right]^2 \right], \quad \text{(S1)}$$

where

$$A^w_{ij} := A_{ij}/f(d_{ij})$$
$$w_{ij} := |f(d_{ij})|^2.$$

With a weighted Euclidean inner product in the space $\mathscr{A}$, $\langle X,Y \rangle = \sum_{\{i,j\}} w_{ij} X_{ij} Y_{ij}$, the problem is equivalent to an l_2-projection of A onto im(grad) and the minimal norm solution s^w is given by,

$$s^w_i = -\frac{1}{N} \sum_j^N w_{ij} A^w_{ij} = -\frac{1}{N} \sum_j^N f(d_{ij}) A_{ij}. \quad \text{(S2)}$$

Figure S1 shows this distance-integrated potential s^w in th case of both London and Tokyo, with a specified form of the distance-deterrence function, $f(d) = \exp(-d_{ij}/D)$. In the case of London, the parameter D was 13.51 km, determined by a linear regression with the formula $A_{ij} = \text{constant} \cdot \exp(-d_{ij}/D)$, using the Euclidean distance d_{ij} between the centroids of the zones. As shown in Figure S1a, the landscape of the distance-integrated potential s^w is similar to that of potential s without distance-integration. Figure S1b shows the scatter plot between s and s^w. In the case of Tokyo, the estimated parameter D was 20.27 km. Figure S1c shows the landscape of the distance-integrated potential s^w. The distance-integration slightly changed the potential in these examples.

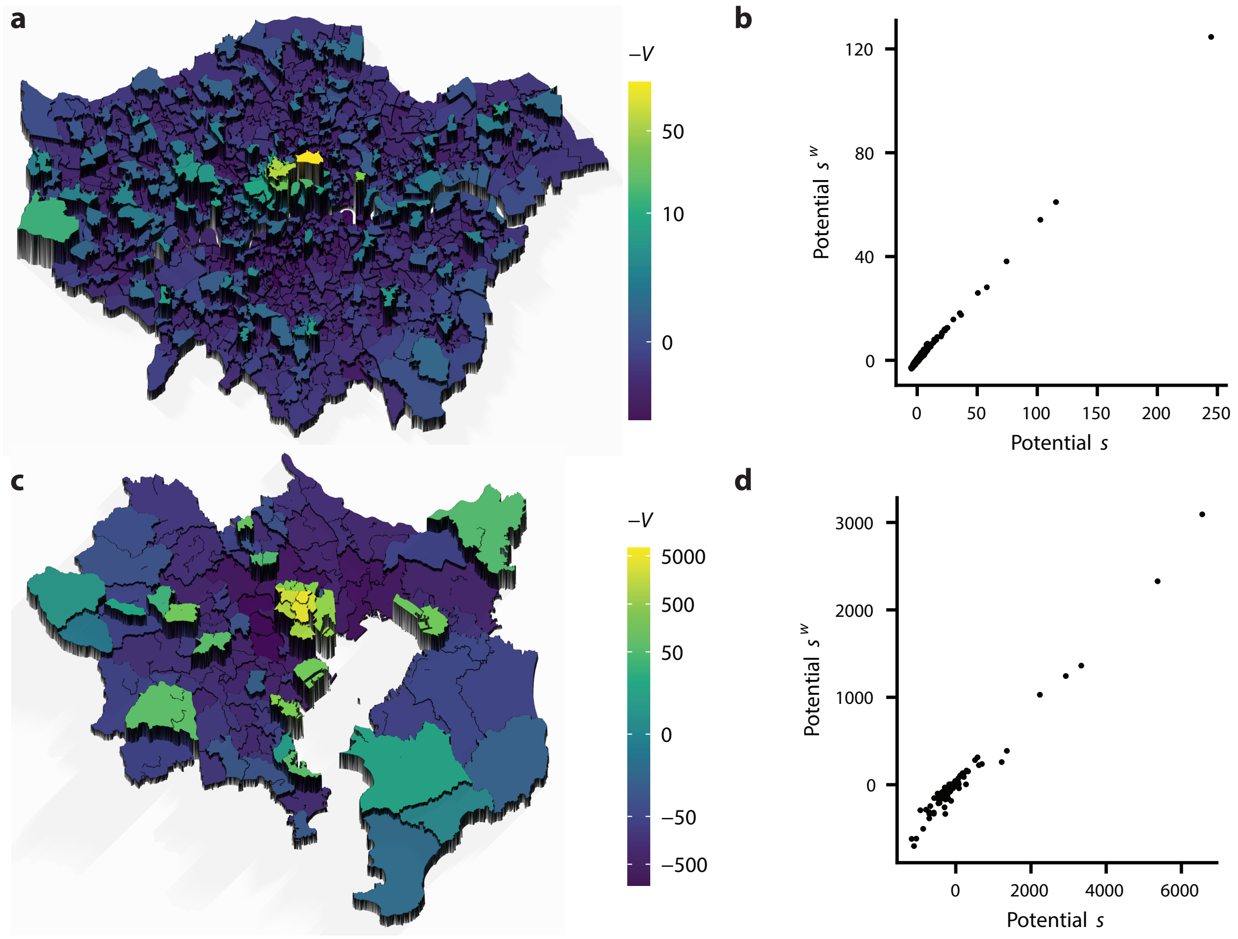

Figure S1. Distance-integrated potential s^w in the London and the Tokyo cases. (**a**) The potential in London is calculated by equation (S2), using the same home-work trip data in Figure 3. (**b**) Scatter plot between the potential without the distance-deterrence effect, s and with the effect, s^w in the case of London. (**c**) The potential in Tokyo is calculated by equation (S2), using the same home-work trip data in Figure 4. (**d**) Scatter plot between s and s^w in the case of Tokyo.
